# Optimization of Ultrasound Pretreatment for Enhanced Drying Efficiency and Piperine Retention in Black Pepper (*Piper nigrum* L.)

**DOI:** 10.3390/foods15010086

**Published:** 2025-12-27

**Authors:** Nana Adwoa Nkuma Johnson, Selorm Yao-Say Solomon Adade, John-Nelson Ekumah, Bridget Ama Kwadzokpui, Turkson Antwi Boasiako, Yi Xu

**Affiliations:** 1 College of Ocean Food and Biological Engineering, Jimei University, Xiamen 361021, China; nana.anjohnson@gmail.com (N.A.N.J.); 202361000120@jmu.edu.cn (Y.X.); 2 Centre for Agribusiness Development and Mechanization in Africa (CADMA AgriSolutions), Ho 00233, Ghana; nelsonjokum@gmail.com; 3 Department of Nutrition and Dietetics, Ho Teaching Hospital, P.O. Box MA 374, Ho 00233, Ghana; 4 School of Food and Biological Engineering, Jiangsu University, Zhenjiang 212013, China; kwadzokpuibridgeta@gmail.com (B.A.K.); turk.sky92@gmail.com (T.A.B.)

**Keywords:** ultrasound pretreatment, response surface methodology, piperine, microstructural characterization, process optimization

## Abstract

Ultrasound pretreatment offers a promising approach for improving spice drying efficiency while preserving bioactive compounds. This study explores the optimization of ultrasound pretreatment parameters for black pepper processing, using response surface methodology (RSM) to maximize piperine retention, drying efficiency, and moisture reduction. Compared to traditional one-factor-at-a-time (OFAT) screening, RSM identifies a multi-objective optimal balance that achieves superior results for all three responses. Our optimized conditions (35 kHz, 40 min, 50 °C, 80 W/cm^3^) achieved 18.64 mg/g DW piperine, a drying time of 444.51 min, and a 9.6% moisture content, demonstrating significant improvements in both bioactive preservation and energy efficiency compared with conventional methods. Compared to control samples requiring 600.69 ± 12.5 min drying time, optimal conditions reduced drying time by 26% to 444.51 min (a net process time reduction of 19%, including a 40 min pretreatment) while achieving the target moisture content (9.6%) and limiting piperine loss to approximately 6% in dried samples. Dual-method validation using UV spectrophotometry and HPLC confirmed model predictions with relative errors below 1%, establishing a consistent UV:HPLC ratio (1:2.12). Multi-analytical characterization revealed that ultrasound-induced cavitation selectively disrupted cellular structures, enhancing mass transfer without significant degradation of piperine’s functional groups. Scanning electron microscopy showed increased porosity and microfractures, while FTIR confirmed preservation of key chemical bonds with minor spectral shifts. The process achieved a five-fold improvement in product consistency (reducing the standard deviation from 0.68 to 0.12 mg/g) compared to conventional drying. These findings demonstrate that optimized ultrasound pretreatment provides a reproducible, scalable, and energy-efficient method for spice processing, supporting industrial adoption where consistent quality and bioactive stability are critical.

## 1. Introduction

Black pepper (*Piper nigrum* L.), often called the “king of spices,” is a globally traded commodity valued for its distinctive flavour, antimicrobial properties, and bioactive compounds, particularly piperine. Beyond its culinary importance, piperine exhibits diverse pharmacological activities, including antioxidant, anti-inflammatory, and bioavailability-enhancing effects [1]. The global spice industry, valued in the billions of dollars, is driven by consumer demand for high-quality products with stable bioactive profiles, making efficient processing and compound preservation essential for commercial viability [2]. While widely adopted in commercial operations, traditional thermal drying methods often compromise bioactive compound integrity due to prolonged heat exposure and inefficient moisture removal, resulting in reduced product quality and economic value [3]. In recent years, ultrasound-assisted processing has emerged as a non-thermal technology capable of reducing mass transfer resistance through cavitation-induced cellular disruption, reducing drying time, and preserving thermosensitive compounds [4]. Acoustic cavitation induces microstreaming, shear forces, and localized pressure gradients, disrupting cellular structures and reducing mass-transfer resistance, thereby facilitating faster moisture removal under milder thermal conditions. Despite the documented benefits, the effective implementation of ultrasound pretreatment for black pepper drying requires systematic optimization of multiple interdependent parameters—including frequency, power density, treatment duration, and temperature—to balance competing objectives of drying efficiency, bioactive preservation, and energy use. Response Surface Methodology (RSM) offers a robust statistical framework for such multi-variable optimization, enabling predictive modelling and interaction effect assessment [5].

Previous studies have applied ultrasound-assisted extraction and drying techniques to various plant materials, demonstrating improvements in mass transfer, drying efficiency, and retention of quality. For example, ultrasound-assisted extraction has been widely used to recover bioactive compounds from plant matrices by enhancing cell disruption and solvent penetration [6], while ultrasound pretreatment has improved drying kinetics and preserved quality attributes in peppers (*Capsicum annuum*) [7] and blood orange slices [8]. Similar enhancements in mass transfer have been observed in habanero chili pepper (*Capsicum chinense*), where ultrasound pretreatment before air drying significantly accelerated moisture removal [9]. However, few have integrated parameter optimization with dual analytical validation and multi-analytical mechanistic characterization. Such integration is crucial for translating laboratory-scale findings into scalable industrial solutions. The present study advances this field by employing a Box–Behnken Response Surface Methodology approach to determine optimal ultrasound conditions for black pepper drying, validating the predictive models through both UV spectrophotometry and HPLC to establish a consistent cross-method correlation, and elucidating the mechanistic effects of ultrasound on piperine stability and matrix structure using FTIR and SEM analyses. This study advances black pepper processing through three original contributions: (1) integrating Box–Behnken RSM optimization with dual analytical validation (UV-HPLC) and establishing a robust cross-method correlation (1:2.12 ratio, R^2^ = 0.995) for rapid process monitoring, (2) combining FTIR and SEM mechanistic characterization to elucidate ultrasound effects on piperine stability and cellular architecture, and (3) quantifying mass transfer enhancement via effective moisture diffusivity modeling that links processing parameters to drying kinetics. Unlike previous single-method studies, this integrated framework systematically validates multi-objective optimization while mechanistically explaining the observed five-fold consistency improvement and 19.3% net process time reduction, providing a reproducible, scalable approach for industrial spice processing where batch-to-batch quality uniformity is critical.

## 2. Materials and Methods

### 2.1. Materials and Reagents

Fresh peppercorns (*Piper nigrum* L.) were procured from commercial suppliers in Hainan, China, and stored at 4 °C under refrigerated conditions to maintain freshness and preserve bioactive compound integrity. Analytical grade piperine standards (≥98% purity) were obtained from Sinopharm Chemical Reagent Co., Ltd. (Shanghai, China) for calibration and quantification protocols. HPLC-grade solvents, including methanol and acetonitrile, were purchased from Merck (Darmstadt, Germany) to ensure analytical precision, while additional chemicals and reagents were of analytical grade from established suppliers. Ultrapure water (18.2 MΩ·cm resistivity) generated using a Millipore Milli-Q purification system (Bedford, MA, USA) was employed for all solution preparations and analytical procedures.

### 2.2. Experimental Design and Optimization Strategy

The investigation employed a two-phase, sequential experimental design adapted from [10] to optimize ultrasonic parameters for peppercorn processing. Initial one-factor-at-a-time (OFAT) screening evaluated four ultrasonic parameters: frequency (20–50 kHz), treatment time (20–60 min), water bath temperature (35–65 °C), and power density (40–140 W/cm^3^), with peppercorns immersed in water at a 3:1 ratio following modified protocols of Johnson et al. (2023) [10]. OFAT screening results were analyzed using one-way ANOVA followed by Tukey’s post hoc test (*p* < 0.05) to identify significant differences among treatment levels. Significant parameters identified during screening were subsequently optimized using Response Surface Methodology via Design-Expert software (Version 13.0.5.0). Subsequently, a Box–Behnken design with four factors at three levels was implemented, comprising 29 experimental runs: 24 factorial points and 5 center-point replications.

The relationship between independent variables and responses was modeled using second-order polynomial equations:

(1) $$Y=\beta_{o}+\sum_{\left(i=1\right)}^{3}\beta_{i}X_{i}+\sum_{i=1}^{3}\sum_{j=i+1}^{3}\beta_{ij}X_{i}X_{j}+\sum_{\left(i=1\right)}^{3}\beta_{ii}X_{i}^{2}$$

where *Y* represents the predicted response, *β*_0_ is the intercept, *β_i_* denotes linear coefficients, *β_i__i_* represents quadratic coefficients, *β_i__j_* indicates interaction coefficients, and *X_i_*, *X_j_* are independent variables.

Multi-response optimization employed the Total Desirability Index:

(2) DI=[∏i=13di(yi)]13

where *d_i_* represents individual desirability indices for piperine content (maximize), drying Time (minimize), and moisture content (target 8–10%).

Optimization prioritized drying efficiency while maintaining acceptable piperine retention, recognizing that economic value derives primarily from reduced processing time and energy consumption rather than from maximizing absolute bioactive content.

#### 2.2.1. Convective Drying Procedure

Following ultrasound pretreatment (or no pretreatment for control samples), peppercorn samples (10 ± 0.01 g) were subjected to hot-air drying in a laboratory convective dryer at 65 °C with an air velocity of approximately 1.0 m/s. Air velocity was estimated based on the oven’s rated power (2.3 kW) and internal dimensions, consistent with reported values for similar laboratory convection ovens [11]. Drying time was determined as the duration required to reach the target moisture content range, monitored gravimetrically at 8 h intervals during the initial drying phase and then at 60 min intervals as samples approached the target moisture content. Drying was terminated when the sample weight stabilized within ±0.1 g over two consecutive measurements, indicating that equilibrium moisture content had been achieved. Final moisture content was verified using the oven method. Dried samples were cooled to room temperature in a desiccator before subsequent analysis. Control samples without ultrasound pretreatment required 600.69 ± 12.5 min to reach the target moisture range under identical drying conditions, serving as the baseline for evaluating the reduction in drying time.

#### 2.2.2. Energy Consumption Analysis and Ultrasound Intensity Quantification

Total energy consumption was evaluated by accounting for ultrasound pretreatment energy, sensible heating energy, and latent heat of moisture evaporation during drying. This approach was adopted to enable a transparent energy balance and facilitate comparison with industrial drying systems.

Ultrasonic pretreatment was conducted using a bath-type ultrasonic processor operating at a fixed machine-set power density of 80 W/L. Ultrasonic energy consumption ($E_{US}$) was calculated as

(3) $$E_{US}=\frac{P_{density}\cdot V_{sample}\cdot t_{US}}{\eta_{trans}}$$

where *P_density_* is power density (W/L), t_US_ is pretreatment time (s), $\eta_{trans}$ is the transducer efficiency (assumed as 0.6 to account for electrical-to-acoustic conversion losses), and $V_{sample}$ represents the total treatment volume. The sample volume (*V_sample_*) was calculated as 0.2 L based on the experimental load of 50 g of black pepper and 150 mL of water (3:1 *w*/*w* ratio), accounting for the displacement volume of the peppercorns (ρ ≈ 1.05 g/cm^3^). Ultrasonic energy was first calculated at the batch scale (50 g) and subsequently allocated proportionally to the 10 g analytical portions used for drying and analysis, ensuring consistency with untreated controls.

Drying energy consumption was estimated based on thermodynamic requirements due to the absence of direct electrical power metering. Total drying energy ($E_{drying}$) was calculated as

(4) $$E_{drying}=\frac{E_{sensible}+E_{latent}}{\eta_{thermal}}$$

where $\eta_{thermal}$ represents the thermal efficiency of the laboratory dryer (assumed as 0.3–0.5). Sensible heating energy was calculated as

(5) $$E_{sensible}=(m_{dry}\cdot 1.5+m_{w}\cdot 4.186)(T_{air}-T_{ambient})\;$$

and latent heat of evaporation as

(6) $$E_{latent}=m_{water\;removed}\cdot \lambda$$

where $m_{dry}\;$ is the dry solid mass, $m_{w}\;$ is the internal water mass of the sample, and λ  is the latent heat of vaporization of water (2260 kJ kg^−1^).

Specific energy consumption (SEC) was calculated as total energy input per kilogram of water removed (kJ kg^−1^), providing a normalized metric suitable for comparison across processing conditions and for scale-up assessment

#### 2.2.3. Mechanistic Modeling of Mass Transfer

To enhance the industrial interpretability of the drying process beyond empirical optimization and to relate ultrasound pretreatment to intrinsic material transport properties, a mechanistic diffusion framework based on Fick’s Second Law was employed. Unlike empirical or semi-theoretical drying constants that depend strongly on dryer configuration, this approach enables estimation of the effective moisture diffusivity ($D_{\mathrm{eff}}$), which reflects the internal mass transfer resistance of the material.

Black peppercorns were approximated as spherical particles, consistent with their natural morphology. The analytical solution for unsteady-state moisture diffusion in a sphere, assuming uniform initial moisture distribution, negligible shrinkage, and negligible external mass transfer resistance (high Biot number), is given by Crank (1975) [12]:

(7) $$MR=\frac{6}{\pi^{2}}\sum_{n=1}^{\infty }\frac{1}{n^{2}}exp\left(-\frac{n^{2}\pi^{2}D_{eff}t}{r^{2}}\right)$$

where MR  is the dimensionless moisture ratio, $D_{\mathrm{eff}}$ is the effective moisture diffusivity (m^2^/s), r is the particle radius (m), and t  is drying time (s).

For extended drying periods in the falling-rate regime (MR<0.6), the infinite series converges rapidly and may be approximated by the first term (n=1), yielding:

(8) $$MR\approx \frac{6}{\pi^{2}}exp\left(-\frac{\pi^{2}D_{eff}t}{r^{2}}\right)$$

To facilitate estimation from experimental drying endpoints, the equation was linearized as

(9) $$ln(MR)=ln\left(\frac{6}{\pi^{2}}\right)-\left(\frac{\pi^{2}D_{\mathrm{eff}}}{r^{2}}\right)t$$

Because time-resolved moisture ratio curves were not collected during drying, $D_{\mathrm{eff}}\;$ was estimated using an endpoint method, based on the total drying time required to reach the final moisture content. The moisture ratio at the endpoint was approximated as

(10) $$MR_{f}\approx \frac{M_{f}}{M_{0}}$$

where $M_{0}$ is the initial moisture content and $M_{f}$ is the experimentally measured final moisture content (wet basis).

The mean particle radius was fixed at $r=2.6\times 10^{-3}$ m (average diameter 5.2 mm), based on reported morphometric characteristics of *Piper nigrum* berries [13]. The initial moisture content was assumed to be 72% (wet basis) [14,15], corresponding to literature values for fresh mature black pepper berries. Using these assumptions, the apparent effective moisture diffusivity was calculated as

(11) $$D_{\mathrm{eff}}=-\frac{r^{2}}{\pi^{2}t}ln\left(\frac{MR_{f}\pi^{2}}{6}\right)$$

The resulting values are reported as apparent effective moisture diffusivities, intended for comparative mechanistic interpretation of mass transfer behavior under different ultrasound pretreatment conditions rather than as definitive diffusivity constants.

### 2.3. Analytical Methodologies

#### 2.3.1. Moisture Content Determination

Moisture content was determined gravimetrically following [16]. Ground peppercorn samples (10 ± 0.01 g) were dried at 105 °C for 24 h. Moisture content was calculated as

(12) $$\%\;\mathrm{Moisture}\;\mathrm{content}=\frac{W2-W3}{W2-W1}\times 100$$

where *W*1, *W*2, and *W*3 represent the weights of the empty crucible, the crucible with the sample, and the crucible with the dried sample, respectively.

#### 2.3.2. Determination of Piperine Content via UV-Spectrophotometry

The piperine content in ultrasound-treated peppercorns was determined using UV-spectrophotometry following a modified protocol based on Patil’s method [17]. To prepare the standard calibration curve, piperine (10 μg/mL) was accurately weighed and dissolved in methanol in 10 mL amber-colored volumetric flasks to protect the solution from light degradation. Working standard solutions at concentrations of 0.5, 1, 2, 4, 8, 16, and 32 μg/mL were prepared through serial dilution of the stock solution. The absorbance of these standard solutions was measured at a wavelength of 342 nm using a UV-spectrophotometer, and a linear regression equation was established by plotting concentration against absorbance. For piperine extraction from the peppercorn samples, freshly ultrasound-treated peppercorns (10 ± 0.01 g) were finely ground to increase the surface area for efficient extraction. A precisely weighed portion of the ground material (10 ± 0.01 mg) was transferred to a flask containing 10 mL of methanol. The mixture was subjected to sonication for 15 min to facilitate the extraction of piperine. The resulting solution was filtered through Whatman filter paper to remove particulate matter. The filtrate was then analyzed for piperine content using the same spectrophotometric method employed for the standard curve. The concentration of piperine in the samples was determined by interpolation using the established linear regression equation from the standard calibration curve.

#### 2.3.3. Determination of Piperine Content via High-Performance Liquid Chromatography

The piperine content of peppercorn samples was determined according to the GB/T 17528-2009 standard method using high-performance liquid chromatography (HPLC) [18]. Peppercorn samples were initially ground into fine powder using a laboratory pulverizer to ensure uniform extraction. Approximately 0.5 g ± 0.0001 g of the powdered sample was accurately weighed and transferred to a conical flask. The active compound was extracted using 50 mL of 95% ethanol as the extraction solvent. The flask was tightly sealed to prevent solvent evaporation, then placed in an ultrasonic bath maintained at 65 °C for 3 h to facilitate complete extraction of piperine. Following the extraction process, the solution was allowed to cool to room temperature before quantitative transfer to a 100 mL volumetric flask. The original conical flask was rinsed several times with small portions of ethanol to ensure complete transfer of the extract, and these rinses were added to the volumetric flask. The solution was then diluted to volume with ethanol and thoroughly mixed to ensure homogeneity. The solution was subsequently filtered to remove any particulate matter that could interfere with the chromatographic analysis.

For HPLC analysis, a 1 mL aliquot of the filtrate was pipetted into a 25 mL volumetric flask and diluted to volume with ethanol to achieve an appropriate detection concentration. The chromatographic analysis was performed using an Agilent 1100 HPLC system (Santa Clara, CA, USA) equipped with a diode array detector (DAD). Separation was achieved on an Agilent TC-C18 column (Santa Clara, CA, USA) (250 mm × 4.6 mm, 5 μm particle size) maintained at 40 °C. A gradient elution program was employed as the mobile phase to facilitate optimal separation of piperine from other compounds. The detection wavelength was set to 210 nm, corresponding to the maximum absorption of piperine. The flow rate was maintained at 1 mL/min, and a 10 μL injection volume was used for each analysis. The piperine content was quantified using an external standard calibration method.

The UV spectrophotometric and HPLC methods were validated for linearity and sensitivity parameters, including the limits of detection (LOD) and quantification (LOQ). Calibration curves were established across the working concentration ranges, and regression analysis confirmed strong linearity. The LOD and LOQ were calculated from the calibration data using the standard deviation of the response (σ) and the slope (S) of the calibration curve, according to ICH guidelines (LOD = 3.3σ/S; LOQ = 10σ/S) [19]. Method precision was assessed using the relative standard deviation (%RSD), with an acceptance criterion of ≤5.0%. All piperine results were expressed in mg/g DW.

### 2.4. Microstructural and Chemical Characterization

#### 2.4.1. Scanning Electron Microscopy (SEM) Analysis

The microstructural characteristics of ultrasound-treated peppercorns were examined using scanning electron microscopy (SEM) following a modified method of Janiszewska-Turak et al. [20]. Peppercorn samples were mounted on copper specimen holders using double-sided adhesive tape and sputter-coated with a thin layer of gold (approximately 10 nm) under vacuum conditions to enhance conductivity. Micrographs were captured using a Hitachi S-3400N scanning electron microscope (Hitachi High Technologies, Tokyo, Japan) at an acceleration voltage of 2 kV. Multiple fields of view were examined to ensure representative imaging of the samples’ surface morphology and structural features.

#### 2.4.2. Fourier Transform Infrared (FTIR) Spectroscopy

**Figure 1:**
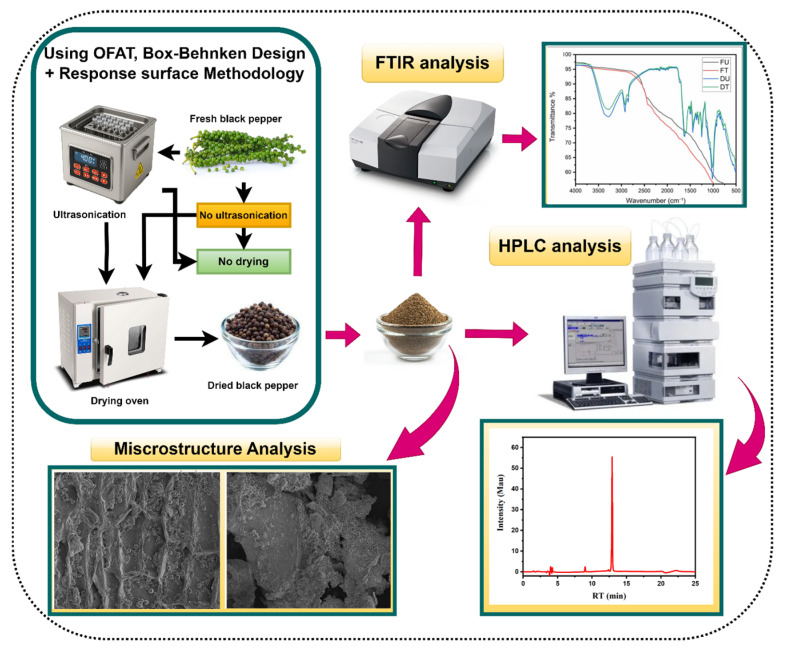
Schematic representation of the experimental workflow.

Chemical characterization of the peppercorn samples was performed using Fourier transform infrared (FTIR) spectroscopy, following the method of Deng & Huang (2025) [21] with minor modifications. Freeze-dried peppercorn samples (4 mg) were finely ground with an agate mortar and pestle and homogeneously mixed with 200 mg of spectroscopic grade potassium bromide (KBr, previously dried at 105 °C for 24 h). The mixture was compressed into transparent pellets (1–2 mm thickness) using a hydraulic press under 10 tons of pressure. Spectral data were collected using a Nicolet IS50 spectrophotometer (Thermo Nicolet Corporation, Madison, WI, USA) in the mid-infrared region (4000–400 cm^−1^) at a resolution of 4 cm^−1^ with 128 scans per spectrum. A blank KBr pellet was used as a reference for background subtraction. After ultrasonic treatment, the resultant spectra were analyzed to identify characteristic functional groups and structural changes in the peppercorn samples. The overall experimental workflow, including ultrasound pretreatment, drying, and subsequent analytical characterization, is illustrated in Figure 1.

#### 2.4.3. Statistical Analysis and Model Validation

Statistical analysis employed multiple software platforms for comprehensive data interpretation and model validation. Response surface methodology and optimization were performed using Design-Expert Software version 11.0.5.0 (STAT-EASE, Inc., Minneapolis, MN, USA), with model adequacy assessed through ANOVA, lack-of-fit tests, predicted versus actual plots, coefficient of variation, and coefficient of determination at *p* < 0.05, *p* < 0.01, and *p* < 0.001 significance levels. Graphical analysis was performed using Prism Software Version 9 (GraphPad, Inc., San Diego, CA, USA), while comparative analysis employed multiple t-tests and Tukey’s post hoc testing at *p* < 0.05 using OriginPro software Origin (Version 2019b, Microcal Inc., Northampton, MA, USA). All experiments were conducted in triplicate with results expressed as means ± standard deviations, and multivariate statistical techniques identified correlations between ultrasonic parameters and physicochemical properties.

## 3. Results

### 3.1. One-Factor-At-a-Time (OFAT) Parameter Screening

**Figure 2:**
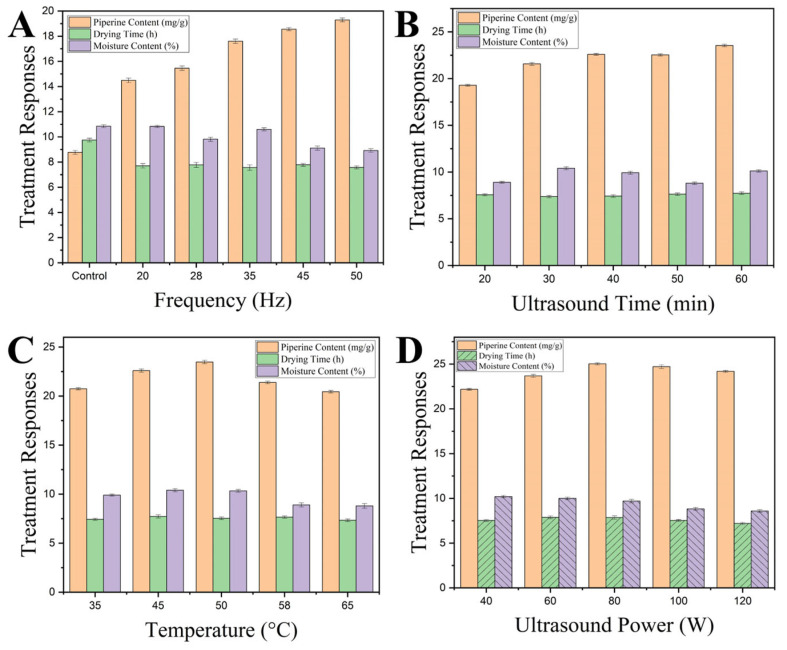
Screening results showing preliminary optima (OFAT): Effect of (**A**) frequency (20–50 kHz), (**B**) treatment time (20–60 min), (**C**) water bath temperature (35–65 °C), and (**D**) power density (40–120 W/cm^3^) on piperine content (orange bars, mg/g DW), drying time (green bars, hours), and moisture content (purple bars, %). Each parameter was varied independently while holding others constant at baseline conditions. Data represent mean ± SD (n = 3). Optimal conditions: 50 kHz, 40 min, 50 °C, 100 W/cm^3^ (determined by UV spectrophotometry), serving as input boundaries for RSM optimization.

The comprehensive one-factor-at-a-time screening systematically evaluated four ultrasonic parameters across their respective ranges, summarized in Figure 2 and Table S1. The study demonstrated distinct parameter-dependent responses across all four ultrasonic variables, revealing complex interactions between processing conditions and both drying efficiency and bioactive compound preservation. These findings provided critical insights into the mechanisms governing the effectiveness of ultrasonic pretreatment in black pepper processing. The piperine concentration values reported in this section were determined using UV spectrophotometry to enable rapid screening across multiple experimental conditions. This analytical approach provided sufficient precision for identifying optimal parameter trends and ranges, facilitating efficient screening of the four-parameter optimization space while maintaining practical experimental timelines.

Frequency screening evaluated five levels (20, 28, 35, 45, and 50 kHz), confirming 50 kHz as the optimal condition with maximum piperine content (19.29 mg/g DW), concurrent with the minimum drying time (7.56 h) and the lowest moisture content (8.9%) (Figure 2A). This frequency-dependent enhancement can be attributed to the fundamental relationship between frequency and cavitation effects. At higher frequencies, cavitation bubbles become smaller but more numerous, creating more uniform and gentler mechanical effects throughout the plant matrix [4]. This contrasts with lower frequencies, which generate fewer but larger cavitation bubbles that collapse more violently, potentially damaging sensitive bioactive compounds [22]. Interestingly, while frequency significantly affected piperine retention, its impact on drying time was minimal, indicating that frequency primarily influences the cellular-level modifications that affect bioactive compound stability rather than bulk moisture movement.

Building on the frequency-optimization findings, the treatment-time effects revealed a complementary temporal dimension to ultrasonic processing optimization. Treatment time optimization showed peak performance at 40 min, yielding a piperine content of 22.59 mg/g DW and a corresponding drying time of 7.43 h. Extended treatment beyond 40 min resulted in plateauing, with minimal improvement in any measured parameters. Treatment durations below 30 min were associated with reduced effectiveness across piperine and moisture responses (Figure 2B). The time-dependent response pattern demonstrated initial rapid improvements followed by a plateau, consistent with two-stage extraction kinetics reported in the ultrasonic processing literature. Yilmaz et al. (2025) [8] described a similar biphasic behavior, in which initial rapid cell wall disruption facilitated bioactive release, followed by equilibrium between extraction and potential degradation processes. The moderate reduction in drying time observed with extended treatment duration suggested that prolonged ultrasonic exposure primarily enhanced cellular permeabilization rather than creating additional moisture-migration pathways. Radziejewska-Kubzdela et al. (2023) [23] further demonstrated that the effects of ultrasonic pretreatment on moisture diffusivity reached saturation after sufficient cellular modification, explaining the observed plateau in the response at extended treatment times.

The temperature parameter introduced thermal considerations that significantly influenced the balance between processing efficiency and bioactive preservation. Temperature screening identified 50 °C as optimal, producing piperine retention of 23.47 mg/g DW with drying time of 7.53 h. Temperatures above 50 °C led to a decline in piperine content. Lower temperatures (35–45 °C) reduced processing efficiency without compensating for the loss of bioactive benefits (Figure 2C). The temperature-dependent response pattern reflected competing mechanisms between enhanced mass transfer at elevated temperatures and potential oxidative processes or altered extraction equilibria, as 50 °C is insufficient for direct thermal degradation of piperine. Briars & Paniwnyk (2012) [24] reported similar temperature optima in bioactive extraction, where moderate heating enhanced solvent penetration and reduced viscosity while avoiding thermal decomposition of target compounds. The declining piperine content observed at temperatures above 50 °C likely reflects enhanced oxidative processes or altered extraction equilibria rather than direct thermal degradation, as piperine typically shows thermal stability at temperatures well above 50 °C [1]. The temperature-dependent response may result from changes in solvent properties, altered cellular matrix behavior, or modified cavitation effects at elevated temperatures. The simultaneous increase in drying time at higher temperatures suggested that excessive heating might compromise cellular structural integrity, reducing the effectiveness of ultrasonic cavitation for enhancing moisture removal. The temperature optimum at 50 °C reflects a balance between beneficial effects (reduced viscosity, enhanced molecular mobility, improved cavitation bubble dynamics) and potential detrimental effects (altered membrane integrity, modified protein-alkaloid interactions, or enhanced oxidative conditions). This relatively low temperature optimum suggests that the observed effects are primarily physical rather than thermal-chemical in nature.

Complementing the frequency, Time, and temperature optimization results, power density screening provided insights into energy requirements for optimal processing outcomes. The investigation identified 100 W as optimal for piperine retention (24.71 mg/g DW), while drying efficiency showed minimal variation across the tested range (40–120 W/cm^3^), with drying times ranging from 7.2 to 7.9 h (Figure 2D). The observed power-dependent response aligns with the mechanistic explanation proposed by Bhargava et al., (2021) [25], wherein moderate acoustic energy levels (60–100 W) create sufficient pressure variations to enhance mass transfer and modify cellular structures without causing extensive damage. The bell-shaped response curve clearly shows that 100 W (22.5 μg/mL) represents the optimal power density for piperine retention, which aligns with findings by Tiwari (2015) [22], who described the relationship between ultrasonic intensity and extraction efficiency as non-linear due to competing mechanisms. At lower power levels, insufficient acoustic energy fails to generate adequate cavitation. In comparison, at higher power levels (>100 W), the violent collapse of cavitation bubbles generates localized extreme conditions that damage sensitive bioactive compounds. While piperine content peaks at moderate power levels, moisture content declines consistently with increasing power (from 10.3% at 40 W to 8.6% at 120 W), reflecting the enhanced mass transfer effects of more intense acoustic cavitation. However, the relatively modest improvement in moisture removal between 100 W and 120 W, compared with the significant decline in piperine retention, suggests that power levels above 100 W offer diminishing returns.

Collectively, the OFAT results demonstrated that ultrasonic pretreatment optimization requires balancing multiple competing mechanisms, including cavitation-induced cellular disruption, thermal effects, oxidative stress, and improvements in the mass transfer coefficient via microstructural modifications. The identification of optimal conditions (50 kHz, 40 min, 50 °C, 100 W) provided evidence that coordinated parameter selection could achieve synergistic effects, simultaneously optimizing bioactive preservation and processing efficiency. These findings established critical boundaries for response surface methodology optimization, in which interaction effects among the identified optimal parameters were investigated to develop comprehensive mathematical models for industrial process design. The parameter ranges identified through OFAT screening ensured that subsequent RSM experiments would focus on the most promising operating space for achieving the study’s dual objectives of energy-efficient drying and bioactive compound preservation.

### 3.2. Response Surface Methodology and Box–Behnken Design Optimization

#### 3.2.1. Box–Behnken Design Implementation and Model Development

The implementation of Response Surface Methodology using the Box–Behnken Design (RSM-BBD) provided a systematic, statistically robust framework for optimizing ultrasound pretreatment parameters while simultaneously evaluating their effects on piperine retention, drying efficiency, and moisture reduction in black pepper processing. The BBD approach (Table S2) was selected for its efficiency in modeling quadratic response surfaces with reduced experimental runs compared to full factorial designs, while avoiding extreme experimental conditions that could potentially damage the bioactive compounds or create unfeasible processing scenarios [26]. This three-level factorial design systematically evaluated the interactive effects of frequency (20–50 kHz), treatment duration (20–60 min), temperature (35–65 °C), and power density (40–120 W/cm^3^) across 29 experimental runs, enabling comprehensive characterization of the ultrasound parameter space while maintaining experimental efficiency. The UV-based analytical approach during RSM optimization enabled rapid determination of piperine content, providing sufficient analytical precision for mathematical modeling and statistical optimization while maintaining experimental efficiency. The consistent parameter trends identified through UV analysis were subsequently validated using HPLC for definitive quantification in the final validation studies.

The inclusion of moisture content as a third response variable alongside piperine retention and drying time addresses a critical quality control requirement that distinguishes this optimization from simple process efficiency studies. While drying time reduction represents the primary process objective, final moisture content determines product shelf stability, microbial safety, and commercial viability. The 8–10% moisture target was established based on regulatory requirements for shelf-stable dried spices, where moisture content below 10–15% inhibits bacterial growth by reducing water activity below 0.85, the threshold for most pathogenic bacteria [27,28]. For black pepper specifically, moisture content in the range of 8–12% corresponds to water activity below 0.60, effectively preventing the growth of bacteria, yeasts, and molds, ensuring long-term storage stability without refrigeration [28,29]. Modeling moisture content as a response variable, rather than simply monitoring it as a process outcome, enabled simultaneous optimization of all three quality attributes through the desirability function approach, ensuring that accelerated drying through ultrasound pretreatment achieved the required moisture endpoint without over-drying (which wastes energy and degrades texture) or under-drying (which compromises microbial stability). The predictive moisture content model (R^2^ = 0.9941, Table 1) confirmed that ultrasound parameters can be adjusted to achieve target moisture levels consistently, demonstrating the process control capability essential for industrial implementation, where batch-to-batch moisture variability directly impacts shelf life and regulatory compliance.

The developed second-order polynomial models demonstrated exceptional predictive capability across all response variables, with coefficient of determination (R^2^) values of 0.9773 for piperine content, 0.9999 for drying Time, and 0.9941 for moisture content. These high R^2^ values, coupled with adjusted R^2^ values exceeding 0.93 and predicted R^2^ values above 0.82, confirmed the models’ robust predictive capacity and their ability to describe the complex relationships accurately [5] between ultrasound parameters and processing outcomes (Table 1). The adequate precision values, ranging from 23.69 to 472.65, significantly exceeded the threshold of 4.0, indicating sufficient signal-to-noise ratios for reliable navigation of the design space and confirming model adequacy for optimization [30]. The low coefficient of variation values (0.04–0.75%) further validated the precision and reproducibility of the experimental methodology [31], essential characteristics for industrial process development and scaling applications.

Analysis of variance revealed that all linear terms significantly influenced the response variables (*p* < 0.05), with frequency and power density exhibiting the most pronounced effects across all responses. The frequency parameter demonstrated the most substantial individual impact on piperine retention (F-value = 102.17, *p* < 0.0001), consistent with established mechanisms in which lower frequencies (20–35 kHz) generate more intense cavitation bubbles that enhance cellular disruption and mass transfer while potentially causing greater mechanical stress on bioactive compounds [4]. Power density emerged as the primary determinant of drying efficiency (F-value = 16,265.60, *p* < 0.0001), reflecting the direct relationship between acoustic energy input and moisture transport enhancement through increased cell membrane permeabilization and microstructural modifications. Temperature effects were particularly significant for moisture reduction (F-value = 738.91, *p* < 0.0001), indicating the synergistic interaction between thermal and acoustic energy in accelerating water migration and evaporation.

The quadratic terms demonstrated significant curvature effects across all responses, confirming the appropriateness of second-order modeling and indicating that optimal conditions existed within the experimental domain rather than at the boundary conditions. Interaction terms revealed complex parameter interdependencies, with the frequency-power density interaction (AD) exhibiting the most substantial effect across all responses, particularly for piperine content (F-value = 125.55, *p* < 0.0001) and drying Time (F-value = 42,717.22, *p* < 0.0001). This interaction suggests that optimal processing conditions require coordinated adjustment of acoustic frequency and power density to balance the benefits of cellular disruption with the preservation of bioactive compounds, supporting the multi-objective optimization approach employed in this study.

**Figure 3:**
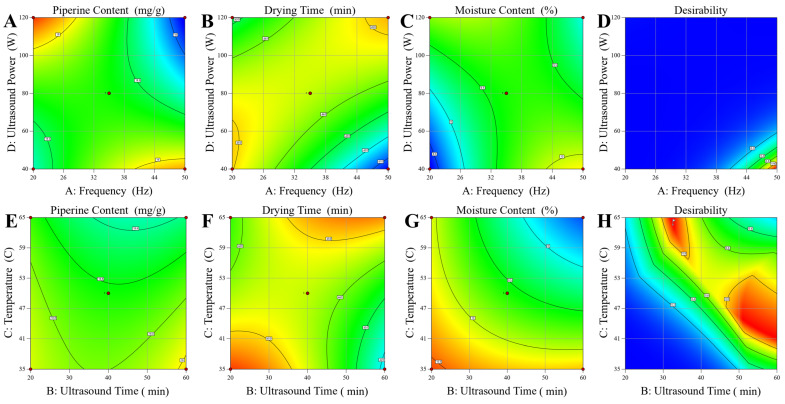
Response surface optimization showing parameter interactions. Frequency × Power interactions for (**A**) piperine content, (**B**) drying Time, (**C**) moisture content, (**D**) desirability. Time × Temperature interactions for (**E**–**H**) same responses. Optimal conditions (●) demonstrate successful multi-objective optimization, achieving maximum desirability through coordinated parameter selection.

Response surface analysis through contour and three-dimensional plots provided visual interpretation of parameter interactions and facilitated identification of optimal processing regions (Figure 3). The frequency-power density interaction plots revealed distinct optimal zones where piperine retention was maximized while maintaining efficient drying performance. Lower frequencies (20–35 kHz) combined with moderate power densities (80–100 W/cm^3^) were favorable for bioactive preservation, while slightly higher power densities were required for optimal drying efficiency. Temperature-time interactions demonstrated that moderate processing conditions (45–55 °C, 30–45 min) provided the best compromise between processing efficiency and compound stability, avoiding excessive thermal stress that could accelerate piperine degradation through oxidative or hydrolytic pathways.

Multi-objective optimization using desirability function analysis successfully identified optimal processing conditions that balanced competing objectives of maximizing piperine retention while minimizing drying time and final moisture content. The optimal conditions were determined as frequency 35 kHz, treatment duration 40 min, temperature 50 °C, and power density 80 W/cm^3^, yielding a composite desirability of 0.847. These conditions predicted a piperine content of 18.63 mg/g DW, a drying Time of 444.51 min, and a final moisture content of 9.6%, representing a favorable balance between bioactive preservation and processing efficiency compared to conventional drying methods. The RSM-derived optimal conditions (35 kHz, 80 W/cm^3^) differ from individual OFAT optima (50 kHz, 100 W/cm^3^) because RSM considers simultaneous parameter interactions and multi-response objectives that single-factor screening cannot capture. While OFAT identified conditions that maximize individual responses, RSM optimization achieved the best compromise across all three responses (piperine retention, drying time, and moisture content) while accounting for complex parameter interactions. This demonstrates the advantage of RSM in identifying global optima that balance competing objectives rather than maximizing individual parameters independently.

**Figure 4:**
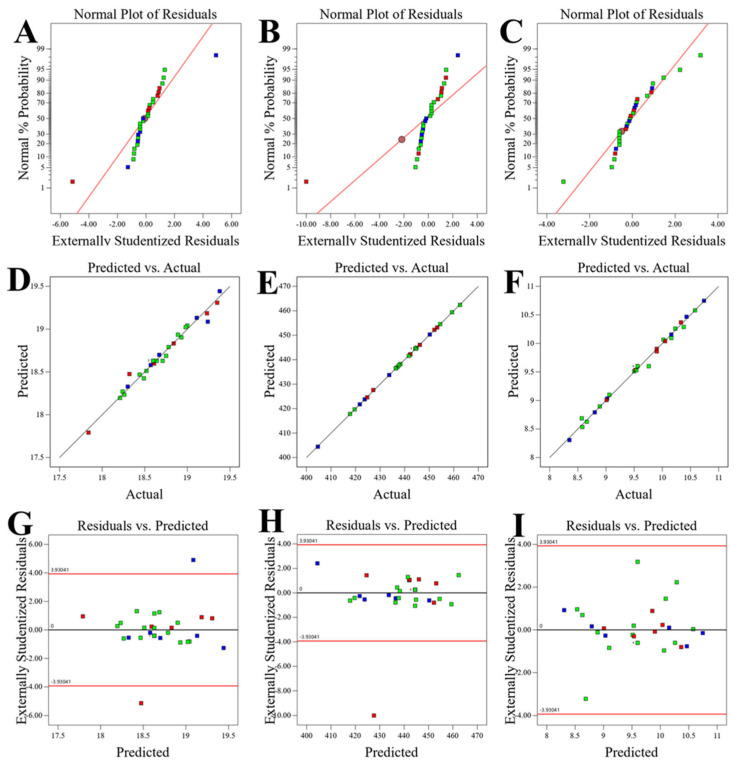
Statistical validation of RSM models. (**A**–**C**) Normal probability plots of residuals. (**D**–**F**) Predicted vs. actual values (R^2^ > 0.96). (**G**–**I**) Residuals vs. fitted values confirming model adequacy.

Statistical validation via diagnostic analysis confirmed the model’s adequacy and reliability (Figure 4). Normal probability plots of residuals (Figure 4A–C) demonstrated approximately normal distribution for all responses, validating the assumption of normally distributed errors essential for ANOVA validity. The diagnostic showed residuals closely following straight lines, confirming normal error distribution, a fundamental requirement for valid regression analysis, and suggesting the absence of systematic bias in the model, a critical assumption for valid statistical inference in RSM [30,32]. Predicted versus actual plots (Figure 4D–F) demonstrated strong alignment between experimental and predicted values, with data points evenly distributed along the 45° line, further validating model accuracy. Finally, the residuals versus fitted values plots revealed random distribution patterns without evident trends, confirming homoscedasticity and model adequacy [33]. The non-significant lack-of-fit test results (*p* > 0.05) for all responses further validated that the quadratic models adequately described the experimental data, without significant lack of fit, supporting their use for optimization and prediction.

#### 3.2.2. Multi-Objective Optimization and Desirability Analysis

The initial One-Factor-At-a-Time (OFAT) screening provided essential insights into general parameter trends and established operational boundaries for subsequent RSM optimization. While OFAT approaches are simple and widely used for preliminary investigations, they cannot capture parameter interactions or identify global optima due to their inherent limitation of varying only one factor while holding others constant [34,35]. Individual screening revealed peak performance for single responses at specific, high-intensity conditions. For instance, frequency screening suggested 50 kHz was optimal for maximizing piperine content (19.29 mg/g DW) and minimizing drying time (7.56 h or 453.6 min), while power density screening identified 100 W/cm^3^ as optimal for piperine retention (24.71 mg/g DW). This preliminary analysis, however, revealed a critical inherent trade-off: conditions that maximize a single response often operate near instability boundaries, potentially sacrificing overall consistency or energy efficiency. Such trade-offs between quality, drying time, and energy inputs are commonly observed in ultrasound-assisted drying and other emerging pretreatment technologies [36]. Crucially, the OFAT experiments served solely as an efficient method for defining the appropriate experimental range for subsequent Box–Behnken Design RSM, consistent with established optimization study standards where OFAT screening precedes statistically rigorous multi-factorial optimization [37]. Rigorous statistical analysis was reserved for the formal RSM models. The control process, which received no ultrasound pretreatment, had an estimated drying time baseline of 600.69 min (10.01 h), which serves as the reference for calculating process efficiency improvements. While ultrasound pretreatment reduced the drying phase by 26% (from 600.69 min in the control to 444.51 min in the treated), the complete process timeline must account for the 40 min pretreatment. The total process time for ultrasound-assisted drying was 484.51 min (40 min pretreatment + 444.51 min drying) compared to 600.69 min for conventional drying, representing a net reduction of 116.18 min, or 19.3% reduction in total processing time. This net time savings, combined with the demonstrated improvements in process consistency and energy efficiency, confirms the industrial viability of ultrasound pretreatment despite the additional processing step. The 26% drying phase reduction alone translates to significant energy savings due to reduced thermal exposure duration, while the pretreatment phase consumes minimal energy relative to extended convective drying.

A critical distinction exists between the single-factor maximum identified by OFAT and the multi-objective optimum identified by RSM. Although OFAT screening suggested higher absolute piperine content could be achieved at 100 W/cm^3^ (24.71 mg/g DW), the final RSM-validated optimum was chosen at 80 W/cm^3^, yielding a lower absolute piperine concentration of 18.62 mg/g DW. This selection is justified by the strategic priority placed on process stability, quality, and consistency, which are integrated into the Total Desirability Index (DI). The desirability function approach transforms multi-response optimization into a single-objective problem by converting each response into a dimensionless desirability value ranging from 0 (completely undesirable) to 1 (ideal), then combining these values into an overall desirability using the geometric mean [5,35]. The DI approach correctly identified that conditions maximizing a single response may not be commercially viable if they result in high product variability, a fundamental principle in quality control where process consistency often outweighs absolute performance. Under the DI approach, the conditions selected—35 kHz frequency, 40 min, 50 °C, and 80 W/cm^3^ power density—provided the best compromise across all objectives. These optimized conditions met the required final moisture content target (validated at 9.6%, critical for shelf stability and microbial safety) and achieved high drying efficiency while simultaneously stabilizing the product output. The most compelling evidence supporting this choice is the substantial reduction in variability: the standard deviation (SD) of piperine content decreased from 0.68 μg/mL (characteristic of high variability under control or single-factor maxima) to 0.12 μg/mL under optimal RSM conditions. This represents a 5.7-fold improvement in process consistency, validating the use of the DI approach to identify a globally superior, industrially reproducible operating point.

### 3.3. Confirmation Study and Dual-Method Analytical Validation

The confirmation study employed a comprehensive dual-analytical approach to validate the RSM-optimized conditions while establishing analytical correlations essential for process verification. Three replicate experiments conducted under the predicted optimal conditions (35 kHz frequency, 40 min treatment duration, 50 °C temperature, 80 W/cm^3^ power density) demonstrated exceptional agreement between predicted and observed values across all response variables, as determined by UV spectrophotometry and HPLC analysis. This dual-method validation approach aligns with best practices in food process optimization, ensuring model accuracy and practical applicability [5].

The UV-based validation (Table 2 and Table S3) demonstrated exceptional model accuracy across all optimized parameters, with observed piperine content of 18.64 ± 0.15 mg/g DW closely matching the predicted value of 18.63 mg/g DW (0.06% relative error), drying time validation achieving 444.41 ± 8.2 min compared to predicted 444.51 min (0.02% relative error), and final moisture content of 9.53 ± 0.18% versus predicted 9.6% (0.73% relative error). These validation results significantly exceed typical RSM model accuracy standards, where relative errors below 5% are considered excellent in food processing applications [5,10]. The UV spectrophotometry results confirmed the second-order polynomial models’ ability to capture the complex relationships between ultrasound parameters and processing outcomes, with all experimental values falling within the 95% prediction intervals and narrow confidence bands (±0.11 μg/mL for piperine, ±0.12 min for drying Time, ±0.13% for moisture content), demonstrating the high predictive precision critical for process control applications where consistent processing outcomes demand accurate predictive models [21].

Parallel HPLC analysis of identical confirmation samples provided definitive piperine quantification while establishing critical analytical relationships between rapid screening and rigorous validation methods. When normalized to dry weight basis to eliminate moisture as a confounding variable, UV spectrophotometry yielded 18.64 ± 0.12 mg/g DW while HPLC analysis yielded 39.51 ± 0.66 mg/g DW, establishing a consistent UV:HPLC dry weight ratio of 1:2.12 across all confirmation experiments (R^2^ = 0.995). The discrepancy reflects fundamental differences in extraction methodology rather than detector specificity. The UV method employed a small sample mass (10 mg) and a brief room-temperature extraction (15 min of sonication in 10 mL methanol), optimized for rapid screening during the experimental design phases (OFAT and RSM). In contrast, the HPLC method used a larger sample mass (500 mg) and rigorous extraction conditions (3 h at 65 °C in 50 mL ethanol, followed by dilution to 100 mL), designed for exhaustive compound recovery in accordance with GB/T 17528-2009 standard protocols. The observed UV:HPLC ratio of 2.12:1 (dry weight basis) indicates that the UV extraction achieved approximately 47% relative recovery to the standardized HPLC protocol (18.62/39.51 = 0.47), consistent with the less rigorous extraction conditions employed. While UV spectrophotometry at 342 nm may also detect co-eluting alkaloids and phenolic compounds present in black pepper’s complex phytochemical matrix [1], the primary source of the systematic difference is extraction efficiency rather than detector non-specificity. This is evidenced by the UV:HPLC ratio remaining stable across ultrasound treatments (coefficient of variation < 2%), indicating that both methods track relative changes proportionally despite different absolute recovery rates. Critically, when concentrations are normalized to dry weight basis—the chemically appropriate representation for solid samples—HPLC yields substantially higher values (39.51 vs. 18.64 mg/g DW), confirming its superior extraction efficiency and establishing it as the definitive quantification method.

This dual-method validation framework served distinct analytical purposes: UV spectrophotometry enabled efficient, high-throughput screening during OFAT parameter exploration and RSM optimization (29 experimental runs), where relative comparisons and trend identification were paramount. The method’s reproducibility (±0.12 mg/g DW) proved sufficient for mathematical modeling and statistical optimization. Subsequently, HPLC provided absolute quantification for final validation, yielding piperine content of 39.51 ± 0.66 mg/g DW (3.95% *w*/*w*), which falls within the reported literature range for black pepper (2–9% piperine) [1,38] and validates the biological relevance of optimized processing conditions. Following successful validation of RSM models and establishment of UV-HPLC analytical correlations, the subsequent mechanistic characterization focused exclusively on HPLC-derived data, given its superior extraction efficiency and chromatographic specificity. This analytical approach ensures that all mechanistic interpretations regarding piperine stability, structural modifications, and mass transfer effects are based on definitive, exhaustively extracted piperine concentrations rather than the partially extracted values obtained through rapid screening protocols. The HPLC method provides both the extraction completeness and analytical precision necessary to assess ultrasound treatment effects on bioactive compound preservation and processing outcomes. This integrated analytical strategy—combining rapid UV screening for process optimization with rigorous HPLC validation for absolute quantification—represents a practical framework for industrial spice processing where both experimental efficiency and analytical rigor are essential. The consistent UV:HPLC correlation (1:2.12 dry weight ratio, R^2^ = 0.995) enables future studies to employ UV spectrophotometry for cost-effective process monitoring, while periodically validating with HPLC to ensure absolute accuracy. However, the correlation factor should be verified for different pepper varieties, geographic origins, and processing scales to establish broad applicability beyond the single cultivar examined in this study.

### 3.4. Mechanistic Insights and Analytical Validation of Ultrasound Pretreatment for Black Pepper Processing

#### 3.4.1. Mechanistic Synthesis of Ultrasound Effects on Mass Transfer Kinetics and Piperine Stability

The successful application of ultrasound pretreatment for black pepper processing relies on achieving a critical “Balanced Disruption,” a strategic optimization that maximizes drying kinetics (a physical enhancement) while carefully limiting chemical stress to preserve the target bioactive compound, piperine. This strategy, guided by Response Surface Methodology (RSM), led to the selection of optimal conditions (35 kHz frequency and 80 W/cm^3^ power density) that prioritized overall process consistency and throughput over maximizing absolute yield, reflecting paramount industrial priorities [5].

The physical mechanism driving process efficiency is the dramatic reduction in internal mass transfer resistance (R_i_). The moderate acoustic parameters generate stable acoustic cavitation, leading to microstreaming and micro-jets that physically disrupt the cellular structure of the pepper matrix [4]. Scanning electron microscopy confirmed this effect, showing the formation of uniform microcracks and increased porosity, which provide preferred pathways for moisture escape [20]. The microstructural modifications arise from both cavitation effects and the sponge effect, in which ultrasonic waves induce rapid compression and rarefaction cycles, leading to water release through the formation of microscopic channels and intracellular liquid leakage [39,40]. This structural modification successfully accelerated the drying process by 26%, reducing the required time from approximately 600 min to 444.51 min. This kinetic benefit, termed “Kinetic Protection,” is the principal mechanism for preserving quality, as the reduced time under thermal load significantly reduces the opportunity for slow, cumulative degradation pathways that plague conventional drying methods [41]. Furthermore, the highly uniform physical disruption acts as a structural homogenization step, minimizing natural biological variability across batches and leading directly to a five-fold improvement in product consistency (standard deviation reduced from 0.68 to 0.12 μg/mL).

Complementary to the physical acceleration, the chemical mechanism confirms molecular stability under the moderate acoustic regimen. Fourier transform infrared spectroscopy provided definitive evidence that the energy inputs were below the threshold for severe molecular damage, maintaining the core chemical framework of piperine, including the characteristic C=O-N amide (near 1636 cm^−1^) and C=C diene stretching bonds (near 1582 cm^−1^) [1]. The observed minor loss of approximately 6% in absolute piperine concentration is not attributable to severe bulk thermal degradation (given the low 50 °C drying temperature), but is instead hypothesized to result from highly localized oxidative interactions (sonochemical stress) caused by transient radical species generated during cavitation bubble collapse, where localized conditions can reach temperatures up to 5000 K and pressures of 1000 atm [4,42], or from mild structural rearrangements within the newly permeabilized matrix. This interpretation shifts the understanding of degradation away from conventional heat-catalyzed effects towards controlled, localized sonochemical pathways.

In essence, the optimized ultrasound pretreatment functions as a structural catalyst. It modifies the matrix to unlock diffusion pathways (physical), enabling rapid moisture removal (kinetic protection), while concurrently preserving the essential molecular structures (chemical) by carefully managing sonochemical stress. This convergence of physical and chemical evidence validates the technology as an energy-efficient [11,40] and highly reproducible pathway for industrial spice processing.

#### 3.4.2. Analytical Sensitivity and Method Validation

The UV spectrophotometric and HPLC methods were validated for linearity and sensitivity through calibration range, regression analysis, and determination of LOD and LOQ. Both methods exhibited excellent linearity (R^2^ > 0.998) across their respective ranges (Figure S2), with HPLC achieving lower detection limits (LOD: 0.339 µg/mL; LOQ: 1.026 µg/mL) than UV (LOD: 0.473 µg/mL; LOQ: 1.433 µg/mL). Precision was confirmed with %RSD values below 5% (Table 3), highlighting the reproducibility of both techniques. The chromatographic separation of the piperine standard and extracted sample was well resolved, as shown in Figure S1, confirming method specificity and suitability for black pepper matrices.

Our findings are consistent with previous reports. Parab Gaonkar et al. (2022) reported strong linearity and high sensitivity with a QbD-based RP-HPLC method for piperine [19]. In contrast, Kurangi and Jalalpure (2020) achieved even lower LOD/LOQ values (0.015 and 0.044 µg/mL, respectively) with a stability-indicating RP-HPLC method optimized for pharmaceutical formulations [43]. The comparatively higher thresholds in our study are attributable to matrix complexity and calibration design, which were tailored to the concentration range expected under ultrasound-assisted drying rather than to ultra-trace detection. Similar results were reported by Shrestha et al. (2020), who validated HPLC for black pepper samples and obtained comparable sensitivity ranges, reinforcing the applicability of our method for natural food matrices [44].

The UV validation further demonstrates the practicality of spectrophotometry as a rapid screening tool. Patil et al. (2024) showed reliable linearity up to 32 µg/mL (R^2^ = 0.999), whereas our narrower calibration range focused on concentrations most relevant to processing [17]. Despite higher LOD/LOQ values relative to HPLC, the UV method proved sufficiently precise to capture relative treatment-induced changes. The strong correlation between UV and HPLC results (UV:HPLC ratio ≈ 2.36:1, R^2^ = 0.995; Figure S2) indicates that both methods tracked ultrasound-driven changes in piperine content in proportion. Taken together, the dual validation framework—supported by calibration data (Figure S2), chromatographic resolution (Figure S1), and statistical reproducibility (Table 3)—confirms that HPLC provides definitive quantification, while UV offers a cost-effective and reliable alternative for rapid process optimization.

### 3.5. Mechanisms of Ultrasound-Assisted Drying on Piperine Stability, Structural Integrity, and Mass Transfer Enhancement

This study employed HPLC, FTIR, and SEM analyses to explore mechanistic insights into how ultrasound-assisted drying affects piperine stability, structural integrity, and mass transfer. The combined results show that while ultrasound caused modest reductions in absolute piperine content, it preserved the compound’s molecular framework and introduced controlled structural modifications that enhanced permeability and solvent interaction. These effects contributed to greater process reproducibility and reliability compared to conventional drying, and the following sections present these outcomes in detail, focusing on compound stability, microstructural modifications, and enhanced mass transfer coefficient.

#### 3.5.1. Compound Stability

**Figure 5:**
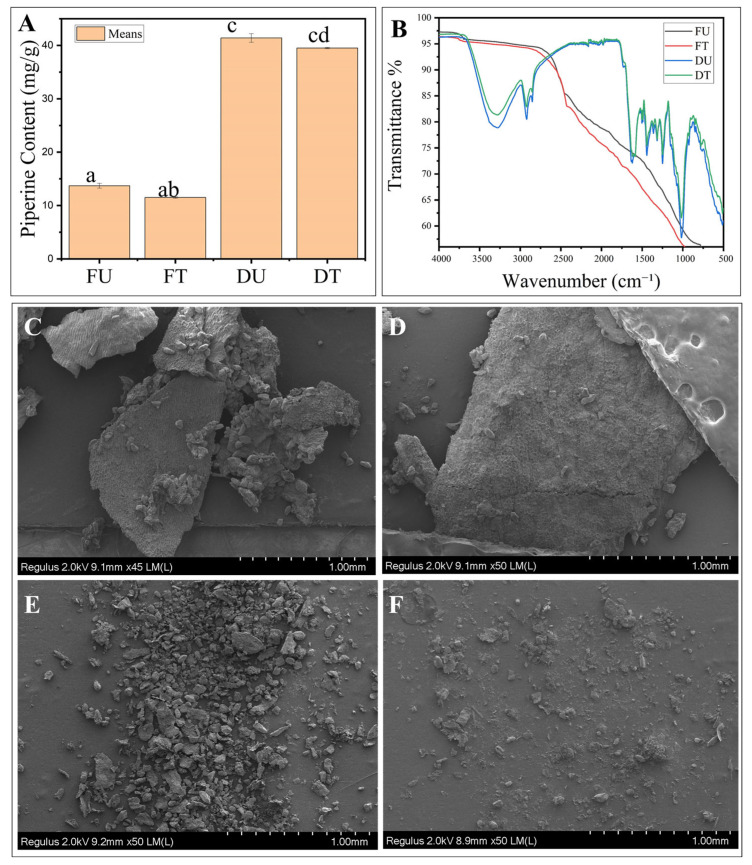
Bioactive compound preservation analysis. (**A**) HPLC piperine quantification showing: fresh untreated (FU: 13.70 ± 0.45 mg/g DW), fresh treated (FT: 11.50 ± 0.14 mg/g DW), dried untreated (DU: 41.80 ± 0.68 mg/g DW), and dried treated (DT: 39.51 ± 0.12 mg/g DW). Different letters (a–d) indicate significant differences (*p* < 0.05, Tukey’s HSD test). Data represent mean ± SD (n = 3). (**B**) FTIR spectra (4000–500 cm^−1^) showing preserved molecular structure after ultrasound treatment: FU (fresh untreated), FT (fresh treated), DU (dried untreated), DT (dried treated). Microstructural SEM images showing (**C**) fresh untreated, (**D**) fresh treated, (**E**) dried untreated, (**F**) dried treated. Scale bars represent 100 μm.

HPLC analysis (Figure 5A) revealed that ultrasound-assisted drying preserved piperine with high consistency, though modest reductions in absolute concentrations were observed compared to conventional drying. The dried ultrasound-treated samples contained 39.51 ± 0.12 mg/g DW of piperine, compared with 41.80 ± 0.68 mg/g DW in untreated dried samples, representing a 5.74% reduction. Fresh samples provided additional perspective, showing 13.70 ± 0.45 mg/g DW in untreated versus 11.50 ± 0.14 mg/g DW in treated, an approximately 16% decrease that likely reflects mild structural rearrangements or oxidative interactions from cavitation effects. Additionally, the piperine reduction is hypothesized to result from localized sonochemical stress during cavitation bubble collapse, where transient hotspots (up to 5000 K) and hydroxyl radicals (•OH) generated in aqueous medium may cause limited oxidative modification of the piperidine ring or amide bond, rather than bulk thermal degradation [42]. Despite these modest losses, ultrasound-treated samples consistently showed very low standard deviations (0.12 for dried, 0.15 for fresh) compared with the much larger variability in untreated dried samples (0.68). This contrast demonstrates that ultrasound effectively stabilizes processing conditions, reducing the influence of uncontrolled factors, such as uneven moisture migration and temperature fluctuations, which typically cause variability in conventional drying [25].

From a process optimization perspective, this balance between slight reductions in compound concentration and significant gains in reproducibility is particularly valuable for industrial applications where consistency and predictability are critical. Ultrasound treatment at moderate frequencies (35 kHz) and durations (40 min) was shown to provide controlled processing conditions, minimizing variability even if localized stresses cause minor molecular modifications. This observation aligns with findings from previous studies, which report that ultrasonic cavitation can induce subtle structural changes in bioactive compounds without disrupting their overall matrix integrity [7,45]. In this study, the five-fold reduction in variability from 0.68 to 0.12 strongly supports the potential of ultrasound as a process control tool, allowing manufacturers to standardize product quality and ensure batch-to-batch reliability, which may be more commercially advantageous than maximizing absolute yields.

Complementary FTIR analysis (Figure 5B) provided further molecular-level evidence that ultrasound treatment preserved the chemical stability of piperine. The major functional bands—such as CO-N stretching and C=C diene stretching near 1636 cm^−1^ and aromatic C=C stretching around 1582 cm^−1^—remained intact, confirming that the essential molecular framework of piperine was not degraded. Additional bands, including the asymmetric stretching of C–O–C (~1250 cm^−1^) and the CH_2_ wagging vibrations (~996 cm^−1^), were also well preserved, reinforcing the compound’s stability [46]. At the same time, subtle shifts such as reduced peak intensities and slight band broadening in the CO-N stretching region suggest that ultrasound introduced limited structural adjustments, likely from localized mechanical and thermal effects, consistent with Shen et al. (2023) [6]. These nuanced changes demonstrate that ultrasound does not destroy sensitive compounds but allows controlled rearrangements within the plant matrix. Taken together with HPLC results, the FTIR evidence confirms that ultrasound-assisted drying achieves a favorable balance—preserving the core molecular features of piperine while introducing process improvements that enhance reproducibility and consistency, thereby strengthening its suitability for practical applications.

#### 3.5.2. Ultrasound-Induced Microstructural Modifications

Building upon the molecular-level findings of piperine stability, the microstructural analysis provided further insight into how ultrasound-assisted drying influences the pepper matrix. SEM micrographs (Figure 5C) revealed that untreated samples maintained a smooth, intact surface with cohesive cell walls, preserving the epidermal structure but limiting access the intracellular compounds. In contrast, ultrasound-treated samples exhibited pronounced porosity, visible microcracks, and ruptured cell boundaries, indicating significant alterations in the cellular matrix. These changes are consistent with the cavitation phenomenon, where collapsing bubbles generate intense microjets that puncture and disrupt plant tissues, thereby creating new pathways for solvent interaction [46]. Such targeted modifications not only help explain the reproducibility observed in piperine stability but also establish ultrasound as a method capable of selectively loosening cellular structures without wholesale destruction of the tissue.

The implications of these morphological changes extend beyond surface disruption to functional advantages in compound accessibility and release. The enhanced porosity and matrix loosening observed in ultrasound-treated samples likely promoted faster and more uniform diffusion of intracellular compounds, including piperine, during subsequent processing. This interpretation is supported by previous studies on fruits and spices, which showed that ultrasound pretreatment increased permeability, reduced mass transfer resistance, and accelerated bioactive compound release [47,48]. In this study, the presence of consistent microfractures and increased surface roughness suggests that ultrasound promotes a controlled form of permeability, enhancing solvent penetration and compound liberation without compromising the overall matrix integrity. Thus, while untreated samples retained their structural cohesion at the cost of reduced accessibility, ultrasound produced a more open and permeable architecture that facilitated more efficient and reproducible compound extraction.

To reinforce these morphological observations, spectral analysis provided additional insight into how ultrasound altered the plant matrix while preserving piperine. FTIR profiles of ultrasound-treated samples revealed that characteristic piperine bands, including CO-N and aromatic C=C stretching, remained intact, confirming chemical stability. At the same time, broader O–H stretching (~3400 cm^−1^), less intense C–H bending (~2920 cm^−1^), and reduced C–O–C stretching (~1030–1060 cm^−1^) pointed to loosening of polysaccharide and cell wall structures, consistent with partial depolymerization effects reported in ultrasound-treated plant systems [7]. Taken together with the SEM evidence, these spectral changes confirm that ultrasound operates through a selective mechanism—weakening the plant cell wall architecture to improve permeability while safeguarding the molecular integrity of piperine. This dual effect underscores the value of ultrasound-assisted drying as a processing approach that achieves enhanced compound accessibility and reproducibility through well-defined structural modifications rather than random degradation. It is important to distinguish that ultrasound does not alter fundamental mass transfer mechanisms (diffusion, convection) but rather reduces mass transfer resistance by modifying the cellular matrix. The observed cellular disruption and increased porosity create preferential pathways that effectively increase the mass transfer coefficient by reducing diffusion path length and eliminating cellular barriers. This represents enhanced mass transfer kinetics through structural modification rather than thermodynamic changes to the transfer driving forces.

#### 3.5.3. Mass Transfer Enhancement and Piperine Yield Increase

Integrating the structural and molecular evidence presented thus far reveals the mechanistic basis for the processing advantages observed with ultrasound treatment. Although ultrasound-treated samples contained slightly less piperine (7.15 ± 0.12 μg/mL) compared to untreated dried samples (7.52 ± 0.68 μg/mL), the dramatic reduction in variability—from a standard deviation of 0.68 to 0.12—highlights its superiority in delivering consistent outcomes. This improvement reflects the cavitation-induced formation of uniform microchannels and increased porosity observed in SEM images, which facilitated more predictable solvent penetration throughout the matrix. FTIR spectra supported this interpretation by revealing controlled loosening of cell wall structures, confirming that ultrasound promoted reproducible mass transfer without compromising the molecular framework of piperine. Together, these results show that ultrasound’s primary advantage lies not in maximizing absolute yield, but in creating reliable processing conditions that ensure reproducible product quality.

Beyond yield considerations, these findings illustrate a shift in processing strategy, where reproducibility and consistency take precedence over absolute concentration. The slight compound losses observed with ultrasound are offset by the systematic improvements in matrix permeability and solvent interaction, which reduce the variability inherent in conventional drying methods. The convergence of evidence from HPLC, FTIR, and SEM highlights ultrasound as a non-destructive approach that enables controlled, repeatable structural modifications, ensuring uniform compound accessibility across samples. Such predictability is particularly valuable in large-scale operations where product uniformity is a key quality parameter. These insights are consistent with earlier reports on ultrasound-assisted processing [6,7,9].

#### 3.5.4. Energy Consumption and Process Scalability Implications

Ultrasound pretreatment significantly reduced drying time by approximately 26%, resulting in a proportional reduction in total drying energy demand. Although the absolute specific energy consumption (SEC) values obtained in this study are elevated due to the use of a laboratory-scale batch pretreatment system, they are not intended to be directly transferable to industrial operation. Instead, the mechanistic energy framework developed here enables parametric translation to industrial systems by substituting equipment-specific parameters such as transducer efficiency, power ratings, loading density, and dryer configuration.

When energy inputs were normalized to the mass of water removed, ultrasound-assisted drying demonstrated improved energy efficiency compared with conventional drying, primarily due to reduced drying time and enhanced mass transfer. Importantly, SEC values were invariant with respect to sample mass when energy allocation was performed consistently, confirming the robustness of the underlying energy balance approach across different batch sizes, even though absolute SEC values will change with scale.

From an industrial perspective, the results indicate that ultrasound pretreatment shifts energy demand from prolonged thermal exposure to short-duration acoustic energy input, which can be more efficiently managed in continuous or hybrid drying systems. In practice, the realized SEC at scale will depend on plant-specific factors, including ultrasound coupling efficiency, spatial distribution of acoustic intensity within larger treatment chambers, and integration with existing dryer throughput and control strategies. Nonetheless, the redistribution of energy input, combined with improved process reproducibility and reduced variability observed at the laboratory scale, provides a quantitative framework to support the engineering design and scalability assessment of ultrasound-assisted drying for spice processing applications.

#### 3.5.5. Kinetic Parameters and Effective Moisture Diffusivity

The effective moisture diffusivity ($D_{\mathrm{eff}}$) quantifies the rate of moisture migration from the interior to the surface of a material during drying. The endpoint-based mechanistic analysis employed in this study revealed that ultrasound pretreatment substantially influenced the internal mass transfer characteristics of black pepper during convective drying. Across the Box–Behnken experimental design (Table S1), the apparent effective moisture diffusivity ($D_{\mathrm{eff}}$) ranged from $3.53\times 10^{-11}$ to $4.34\times 10^{-11}$ m^2^/s, with a mean value of $3.95\times 10^{-11}$ m^2^/s. Higher apparent $D_{\mathrm{eff}}$ values were generally associated with shorter drying times at comparable final moisture contents, indicating reduced internal diffusion resistance. This trend is consistent with the physical role of ultrasound pretreatment in disrupting the pericarp structure, thereby facilitating moisture migration from the interior of the berry to the surface. Using the experimentally validated optimum drying condition obtained from the multi-response RSM model, the apparent effective moisture diffusivity was estimated as $D_{\mathrm{eff},\mathrm{opt}}$ = 3.99 × 10^−11^ m^2^/s. This value lies within the upper range of diffusivities observed across the design space and corresponds to a drying time of 444.41 min and a final moisture content of 9.53%. The agreement between the optimized process conditions and enhanced diffusivity confirms that the RSM-identified optimum represents a physically meaningful improvement in internal mass transfer rather than a purely statistical artifact.

The observed enhancement in apparent diffusivity under ultrasound pretreatment can be attributed to well-established physical mechanisms, including acoustic cavitation and the “sponge effect.” The cyclic compression and rarefaction induced by ultrasonic waves generate microstreaming and localized pressure gradients within the berry matrix, leading to the formation of microscopic channels and fissures in the pericarp [42,49,50]. These structural modifications reduce diffusion path tortuosity and facilitate moisture transport during the subsequent drying stage. The mechanistic findings are consistent with SEM observations showing increased surface roughness and microstructural disruption in ultrasound-treated samples, as well as with the statistically significant reduction in drying time observed across multiple experimental conditions. Although the diffusivity values were derived from endpoint measurements rather than full drying curves, the consistent increase in *D_eff_* relative to non-optimized conditions provides quantitative support for the mass-transfer enhancement induced by ultrasound pretreatment.

The statistically significant reduction in drying time and the corresponding increase in diffusivity observed across experimental conditions strongly support the hypothesis that ultrasound enhances mass transfer by improving the accessibility of moisture within the berry matrix. Although the diffusivity was estimated based on endpoint measurements, which do not capture the dynamic changes in diffusivity over the entire drying process, the consistency of increased $D_{eff}$ values across treatments provides strong evidence of the ultrasound-induced improvement in moisture migration. While the endpoint-based estimation of $D_{\mathrm{eff}}\;$ enables mechanistic comparison across treatments and supports process interpretation, it does not capture potential temporal variation in diffusivity during drying. Future studies incorporating time-resolved moisture measurements will allow more rigorous determination of diffusional parameters and activation energies. Such studies would enable a more detailed exploration of the temporal variation in diffusivity during drying, enhancing our understanding of the drying kinetics and the role of ultrasound in accelerating moisture removal. Nonetheless, the present analysis provides a mechanistic bridge between empirical RSM optimization and physical transport phenomena, strengthening the relevance of the proposed ultrasound-assisted drying strategy for industrial spice processing.

### 3.6. Limitations and Future Directions

Although this investigation provides clear mechanistic and optimization insights into ultrasound-assisted drying of black pepper, it was conducted at the laboratory scale on a single variety, limiting the generalizability of the findings. Prior studies have shown that differences in cultivar, geographic origin, and seasonal variation can significantly influence the bioactive profile and processing behavior of pepper [1]. Similarly, while the focus on piperine allowed precise mechanistic interpretation using HPLC, FTIR, and SEM, black pepper also contains essential oils, alkaloids, and phenolics that contribute to overall quality and may respond differently to ultrasound. Hence, the UV:HPLC correlation established in this study (1:2.12 ratio) requires validation across different black pepper cultivars, geographic origins, and processing conditions before broader adoption for routine process monitoring. While consistent within this study, the systematic difference between methods may vary with matrix composition and warrants investigation in future work to establish method transferability. The current work, therefore, establishes a robust mechanistic foundation while also highlighting the importance of extending investigations beyond a single marker compound and processing context.

Future research should expand toward pilot- and industrial-scale applications to confirm reproducibility and feasibility under commercial conditions. Building on prior calls for a holistic assessment of ultrasound technologies [4], such studies should evaluate the full phytochemical profile of black pepper, as well as its long-term stability, storage behavior, and consumer-relevant quality traits. Integrating techno-economic analysis and life cycle assessment will be critical for demonstrating both financial and environmental benefits, complementing sustainability considerations in modern spice processing. Furthermore, developing continuous-flow ultrasound systems that can be coupled with industrial drying operations, combined with advanced molecular-level characterization tools such as metabolomics and proteomics, would provide deeper insight into structural and functional changes. These steps would not only strengthen the industrial relevance of ultrasound pretreatment but also build upon the mechanistic evidence presented here to establish it as a scalable, reproducible, and sustainable technology for spice processing.

## 4. Conclusions

This investigation optimizes ultrasound pretreatment for black pepper processing by integrating OFAT screening with BBD-RSM, establishing optimal conditions (35 kHz frequency, 40 min duration, 50 °C temperature, 80 W/cm^3^ power density) that outperform conventional thermal drying in process consistency. The study balances bioactive preservation and processing efficiency, achieving optimal piperine retention, drying time, and moisture content through RSM. The combination of UV spectrophotometry and HPLC provides reliable analytical correlations for process monitoring, with validated models offering predictive tools for industrial use. While the results show modest reductions in piperine content (5.74%), a five-fold improvement in process consistency (standard deviation reduced from 0.68 to 0.12 μg/mL) highlights the advantages for commercial applications. Multi-analytical techniques, such as SEM and FTIR, confirm that ultrasound induces controlled cellular disruption and microstructural modifications, enhancing mass transfer while preserving molecular integrity. This study demonstrates that ultrasound pretreatment is an energy-efficient, reliable alternative to traditional drying, with significant potential for industrial adoption. However, further research on scalability, techno-economic feasibility, and the broader phytochemical profile is necessary to realize its full commercial potential.

## Figures and Tables

**Table 1:** ANOVA results, model coefficients, and statistical diagnostic parameters for piperine content, drying time, and moisture content models.

Source	Piperine Content			Drying Time			Moisture Content		
	Mean Square	F-Value	*p*-Value	Mean Square	F-Value	*p*-Value	Mean Square	F-Value	*p*-Value
**Model**	0.2655	43.00	<0.0001	350.07	12,034.67	<0.0001	0.8800	168.74	<0.0001
**Linear**									
A-Frequency	0.3816	61.82	<0.0001	339.52	11,672.21	<0.0001	0.7450	142.86	<0.0001
B-Ultrasound Time	0.0184	2.98	0.1062	414.31	14,243.11	<0.0001	2.48	476.39	<0.0001
C-Temperature	0.5590	90.55	<0.0001	142.21	4888.93	<0.0001	3.85	738.91	<0.0001
D-Ultrasound Power	0.0972	15.75	0.0014	473.14	16,265.60	< 0.0001	0.1220	23.40	0.0003
**Quadratic**									
A^2^	0.1484	24.04	0.0002	51.42	1767.58	<0.0001	1.01	194.48	<0.0001
B^2^	0.2342	37.93	<0.0001	356.20	12,245.58	<0.0001	0.1333	25.55	0.0002
C^2^	0.0146	2.37	0.1459	159.55	5485.10	<0.0001	0.0987	18.92	0.0007
D^2^	0.1687	27.32	0.0001	420.38	14,452.10	<0.0001	0.1048	20.09	0.0005
**Interactions**									
AB	0.0012	0.1984	0.6628	46.31	1591.99	<0.0001	0.0072	1.39	0.2588
AC	0.1560	25.27	0.0002	194.88	6699.70	<0.0001	0.0529	10.14	0.0066
AD	1.35	217.97	<0.0001	1242.56	42,717.22	<0.0001	1.82	349.48	<0.0001
BC	0.0342	5.54	0.0337	890.43	30,611.34	<0.0001	0.3844	73.71	<0.0001
BD	0.4422	71.64	<0.0001	94.28	3241.33	<0.0001	1.09	209.41	<0.0001
CD	0.0056	0.9112	0.3560	16.52	568.08	<0.0001	0.0900	17.26	0.0010
**Diagnosis Statistics**									
Lack of Fit	0.0077	3.36	0.1272	0.0375	4.69	0.0749	0.0041	0.5126	0.8216
R^2^	0.9773			0.9999			0.9941		
R^2^ Adjusted	0.9545			0.9998			0.9882		
R^2^ Predicted	0.8793			0.9995			0.9769		
Adequate precision	29.2220			472.6465			46.9889		
Mean	18.69			439.00			9.60		
C.V. %	0.4203			0.0389			0.7523		
STD	0.0786			0.1706			0.0722		
**Second-order polynomial models for piperine ultrasound parameters**									
Response			Second-order polynomial model						
Piperine Content			18.63 − 0.178333 A − 0.0391667 B − 0.215833 C − 0.09 D + 0.0175 AB + 0.1975 AC − 0.58 AD − 0.0925 BC + 0.3325 BD + 0.0375 CD − 0.15125 A^2^ + 0.19 B^2^ − 0.0475 C^2^ + 0.16125 D^2^						
Drying Time			444.51 − 5.31917 A − 5.87583 B + 3.4425 C + 6.27917 D − 3.4025 AB − 6.98 AC + 17.625 AD + 14.92 BC − 4.855 BD + 2.0325 CD − 2.81542 A^2^ − 7.41042 B^2^ + 4.95958 C^2^ − 8.05042 D^2^						
Moisture Content			9.6 + 0.249167 A − 0.455 B − 0.566667 C + 0.100833 D − 0.0425 AB − 0.115 AC − 0.675 AD − 0.31 BC + 0.5225 BD + 0.15 CD − 0.395417 A^2^ + 0.143333 B^2^ + 0.123333 C^2^ + 0.127083 D^2^						

**Table 2:** Confirmation analysis results at optimal conditions using dual analytical validation.

Parameter	Predicted Mean	UV-Observed Mean ± SD	HPLC-Observed Mean ± SD	Method Correlation
Piperine Content (mg/g)	18.63	18.64 ± 0.11	39.51 ± 0.12	1:2.12 *
Drying Time (min)	444.51	444.41 ± 0.12	444.41 ± 0.12	N/A
Moisture Content (%)	9.6	9.53 ± 0.13	9.53 ± 0.13	N/A

The table includes the confirmed piperine content (μg/mL), drying time (min), and moisture content (%). The final column lists the UV:HPLC concentration ratio which is represented by *, indicating method-specific differences in detection. N/A represents Not Applicable. Data represent mean ± SD (n = 3).

**Table 3:** Validation parameters for piperine determination by HPLC and UV.

Method	Range (µg/mL)	Linear Equation	R^2^	LOD (µg/mL)	LOQ (µg/mL)	%RSD
**HPLC**	0.4–10.0	*y* = 83.6213*x *− 5.3384	0.99949	0.339	1.026	1.14
**UV**	2.0–10.0	*y* = 0.0540*x* + 0.0180	0.99979	0.473	1.433	1.97

## Data Availability

The original contributions presented in this study are included in the article. Further inquiries can be directed to the corresponding author.

## Supplementary Materials

The following supporting information can be downloaded at: https://www.mdpi.com/article/10.3390/foods15010086/s1, Figure S1: Representative HPLC chromatograms of (A) piperine standard (5 µg/mL) and (B) piperine extracted from black pepper sample; Figure S2: Calibration curves for piperine determination using (A) HPLC and (B) UV spectrophotometry at 342 nm; Table S1: OFAT screening results showing individual parameter optima and corresponding responses; Table S2: Box–Behnken design matrix with independent variables and observed responses for ultrasound treatment of black peppercorns. Piperine content was determined by UV spectrophotometry at 342 nm for rapid RSM screening and optimization; Table S3: UV-based confirmation analysis results at optimal conditions.

## Abbreviations

The following abbreviations are used in this manuscript:

RSM	Response Surface Methodology
OFAT	One-Factor-At-a-Time
UV	Ultraviolet (spectrophotometry)
HPLC	High-Performance Liquid Chromatography
FTIR	Fourier Transform Infrared (spectroscopy)
SEM	Scanning Electron Microscopy
BBD	Box–Behnken Design
ANOVA	Analysis of Variance
LOD	Limit of Detection
LOQ	Limit of Quantification
RSD	Relative Standard Deviation
DAD	Diode Array Detector
ICH	International Council for Harmonisation (guidelines)
QbD	Quality by Design
RP-HPLC	Reverse Phase High-Performance Liquid Chromatography
DI	Desirability Index
C.V.	Coefficient of Variation
STD	Standard Deviation
SD	Standard Deviation
kHz	kilohertz
W/cm^3^	Watts per cubic centimeter
μg/mL	micrograms per milliliter
min	minutes
GB/T	Guobiao/Tuijian (Chinese National Standard/Recommended)
KBr	Potassium Bromide
