# Supplementary material for: Optimization of Ultrasound Pretreatment for Enhanced Drying Efficiency and Piperine Retention in Black Pepper (Piper nigrum L.)
Source: Foods. 2025 Dec 27;15(1):86. doi: 10.3390/foods15010086 (PMC12785996; doi:10.3390/foods15010086)
Supplement: Supplementary file 1 [file foods-15-00086-s001.zip › foods-4041069-supplementary.pdf]

Supplementary

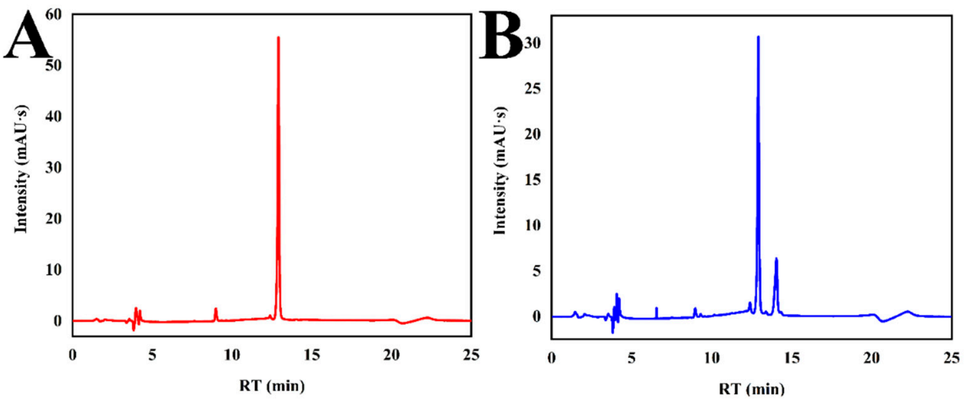

Figure S1. Representative HPLC chromatograms of (**A**) piperine standard (5 μg/mL) and (**B**) piperine extracted from black pepper sample.

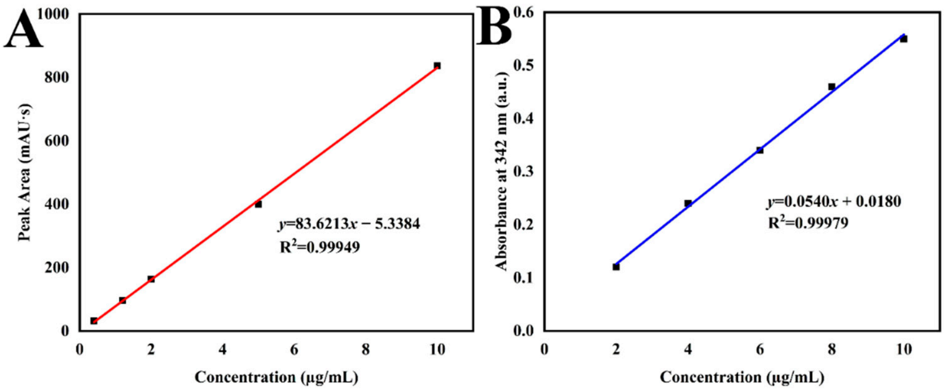

Figure S2. Calibration curves for piperine determination using (**A**) HPLC and (**B**) UV spectrophotometry at 342 nm.

Table S1. OFAT screening results showing individual parameter optima and corresponding responses.

Parameter	Range Tested	Optimal Value	Piperine (mg/g)	Drying Time (h)	Moisture (%)
Frequency (kHz)	20–50	40	22.59 ± 0.11	7.43 ± 0.14	9.92 ± 0.16
Treatment Time (min)	20–60	50	23.47 ± 0.17	7.53 ± 0.13	10.33 ± 0.14
Temperature (°C)	35–65	100	24.71 ± 0.19	7.53 ± 0.11	8.82 ± 0.16
Power Density (W/cm^3)	40–140	40	22.59 ± 0.11	7.43 ± 0.14	9.92 ± 0.16

Data represent mean ± SD (n = 3). Piperine concentrations were determined by UV spectrophotometry for rapid screening and measured in mg/g DW.

Table S2. Box–Behnken design matrix with independent variables and observed responses for ultrasound treatment of black peppercorns. Piperine content was determined by UV spectrophotometry at 342 nm for rapid RSM screening and optimization.

	Independent Variables				Dependent Variables			
Run	Frequency (kHz)	Treatment Duration (min)	Temperature (°C)	Power Density (W/L)	Piperine Content (mg/g)	Drying Time (min)	Moisture Content (%)	
	A	B	C	D	Y1	Y2	Y3	Apparent D_{eff} (m^2/s)
1	35	40	35	40	19.24	433.71	10.43	3.78×10^{-11}
2	35	40	50	80	18.64	444.55	9.76	3.85×10^{-11}
3	20	40	65	80	18.21	462.55	8.66	4.00×10^{-11}
4	35	40	50	80	18.60	444.55	9.56	3.91×10^{-11}
5	50	40	65	80	18.26	437.75	8.89	4.16×10^{-11}
6	35	40	65	120	18.32	453.26	9.52	3.88×10^{-11}
7	35	40	35	120	18.84	442.34	10.33	3.73×10^{-11}
8	20	40	50	120	19.35	427.23	9.90	3.76×10^{-11}
9	50	40	35	80	18.24	444.81	10.23	3.72×10^{-11}
10	35	40	65	40	18.57	436.5	9.02	4.08×10^{-11}
11	35	40	50	80	18.60	444.55	9.56	3.91×10^{-11}
12	50	40	50	40	19.11	404.65	10.16	3.77×10^{-11}
13	50	60	50	80	18.44	419.64	9.06	4.22×10^{-11}
14	20	20	50	80	18.93	442.19	9.50	3.91×10^{-11}
15	35	40	50	80	18.60	444.55	9.56	3.91×10^{-11}
16	20	60	50	80	18.78	437.18	8.57	4.31×10^{-11}
17	35	20	50	120	18.61	446.18	9.90	3.76×10^{-11}
18	35	60	50	120	19.23	424.75	10.05	3.75×10^{-11}
19	35	60	65	80	18.49	454.49	8.58	4.20×10^{-11}
20	35	20	50	40	19.38	423.73	10.74	3.53×10^{-11}
21	20	40	50	40	18.30	450.24	8.35	4.34×10^{-11}
22	35	20	35	80	18.89	459.31	10.58	3.54×10^{-11}
23	35	40	50	80	18.71	444.35	9.56	3.92×10^{-11}
24	35	60	50	40	18.67	421.72	8.8	4.20×10^{-11}
25	50	20	50	80	18.52	438.26	10.16	3.68×10^{-11}
26	20	40	35	80	18.98	441.69	9.54	3.92×10^{-11}
27	50	40	50	120	17.84	452.14	9.01	3.94×10^{-11}
28	35	60	35	80	19.00	417.75	10.38	3.70×10^{-11}
29	35	20	65	80	18.75	436.37	10.02	3.71×10^{-11}

Piperine content was determined by UV spectrophotometry at 342 nm for rapid RSM screening and optimization.

Table S3. UV-based confirmation analysis results at optimal conditions.

**Analysis**	**Predicted Mean**	**Predicted Median**	**Std Dev**	**SE Pred**	**95% PI Low**	**Observed Mean**	**95% PI High**	**Apparent D_{eff} (m^2/s)**
Piperine Content (mg/g DW)	18.63	18.63	0.0714	0.0521	18.45	18.64 ± 0.11	18.81	
Drying Time (min)	444.51	444.51	0.1706	0.1246	444.24	444.41 ± 0.12	444.78	3.99×10^{-11}
Moisture Content (%)	9.63	9.63	0.0722	0.0527	9.49	9.53 ± 0.13	9.71	
